# Association of Underweight and Weight Loss With Poor Prognosis and Poor Therapy Effectiveness in Brain Metastases: A Retrospective Study

**DOI:** 10.3389/fnut.2022.851629

**Published:** 2022-07-01

**Authors:** Yan He, Yu Zhang, Weelic Chong, Yiyan Pei, Renjie Zhang, Zheran Liu, Jiayi Yu, Xingchen Peng, Fang Fang

**Affiliations:** ^1^West China Hospital, Sichuan University, Chengdu, China; ^2^Evidence-Based Medicine Center, Affiliated Hospital of Chengdu University, Chengdu, China; ^3^Department of Medical Oncology, Thomas Jefferson University, Philadelphia, PA, United States

**Keywords:** brain metastases, body mass index–BMI, overall survival (OS), underweight, anti-cancer therapy

## Abstract

**Background:**

The prognostic role of body mass index (BMI) in patients with brain metastases is controversial. We aim to investigate the impact of BMI on prognosis and anti-cancer therapy effectiveness in brain metastases.

**Methods:**

Patients diagnosed with brain metastases between Oct 2010 and July 2019 were followed for mortality through April 2021. The prognostic role of BMI on overall survival was assessed by a restricted cubic spline (RCS) using a flexible model to visualize the relationship between the BMI values and hazard ratios of all-cause mortality, followed by a cox regression model. The disparity of survival outcomes in patients receiving anti-cancer therapies or those did not was evaluated according to the classification of BMI.

**Results:**

A total of 2,466 patients were included in the analysis, including 241 in the underweight (BMI < 18.5 kg/m^2^) group, 1,503 in the normal weight group (BMI 18.5–23.9 kg/m^2^), and 722 in the overweight (BMI ≥ 24 kg/m^2^) group. Relative to the normal weight group, underweight patients were associated with poor prognosis (adjusted HR 1.25, 95% CI 1.07–1.46, *p* = 0.005). However, those in the overweight group showed similar overall survival when compared to the normal-weight group. Patients with weight loss were associated with a higher risk of mortality compared with patients without significant weight loss. In underweight patients, there was an insignificant difference in survival outcomes whether they received anti-cancer therapies or not.

**Conclusion:**

Underweight and significant weight loss were associated with poor prognosis in brain metastases. Meanwhile, anti-cancer therapies did not significantly improve overall survival in patients with underweight. These findings suggest that improving nutrition to maintain body weight is critical for patients with brain metastases.

## Introduction

Brain metastases are detected in approximately 10–40% of patients with cancer (1, 2). Over the decades, the incidence of brain metastases is increasing due to improved imaging techniques and effective systemic treatment of primary cancers (3). Although aggressive therapy has been used, the prognosis is generally poor (4, 5). Several factors were investigated to predict prognosis in these patients, such as age, Karnofsky Performance status, type of primary tumor, and location and number of brain metastases (6). However, the impact of BMI on prognosis in brain metastases was unclear.

The major influence of BMI on cancer prognosis can be rationalized by the effect of fat tissue on hormones and metabolism (7). It was reported that a higher BMI might be advantageous for cancer prognosis because more energy reserves could be drawn on through aggressive treatment (7). Meanwhile, genome expression analysis found that cancer-promoting genes of metabolism and fatty acid presented lower expression in patients with higher BMI (8). However, higher BMI may be associated with worse cancer prognosis *via* increasing serum insulin concentrations and the bioavailability of insulin-like growth factor-I (9). Lean muscle mass is also lost during cancer progression, a phenomenon known as cancer cachexia, with occurrence of other metabolic derangements (10). The complex relationship between BMI and cancer, including brain metastases, remains poorly understood.

In brain metastases, a retrospective analysis including 624 patients with brain metastases reported that the median overall survival of underweight patients was 3 months, which was significantly shorter than healthy or patients who are overweight/obese (7–8 months, *p* < 0.001) (11). Lareida et al. evaluated the correlation of BMI with survival outcomes in brain metastasis, and demonstrated that overweight was associated with better outcomes, while underweight associated with worse outcomes (12). However, another study identified that BMI ≥ 25 kg/m^2^ had a negative impact on overall survival compared with BMI < 25 kg/m^2^ (median overall survival: 13.7 vs. 30.6 months, *p* < 0.001) (13). Whether BMI is a significant predictor of prognosis in brain metastases remains controversial.

Here, we examined whether BMI is a prognostic factor in patients with brain metastases. We performed a retrospective analysis based on 2,466 patients with brain metastases to identify the impact of BMI and weight change on prognosis and to evaluate the disparity of survival outcomes in patients receiving anti-cancer therapies or those who did not according to the classification of BMI.

## Materials and Methods

### Patients and Data Collection

We retrospectively collected data from West China hospital between Oct 2010 and July 2019. The last follow-up time was April 2021. The survival status of patients was also used in the household registration system in China. Patient consent was waived by the Institutional Review Board because no intervention was given, and no patients’ privacy was leaked. To be included in this study, patients had to be pathologically confirmed to have cancer and had radiologic findings of brain metastases. Patients were excluded if they had neoplastic meningitis or were age < 18 years old.

Body mass index was calculated as weight (kg) divided by height squared (m^2^). The first BMI record was assessed when brain metastases were diagnosed. Subsequently, BMI was assessed every 8 weeks to collect weight change data. Patients were divided into three different BMI categories according to the guidelines for prevention and control of overweight and obesity in Chinese adults: underweight group (<18.5 kg/m^2^), normal-weight group (18.5–23.9 kg/m^2^), and overweight or obese group (≥24 kg/m^2^) (14). After brain metastases diagnosis, patients with BMI decreasing by ≥ 1 kg/m^2^ were regarded as having significant weight loss. If BMI decreased < 1 kg/m^2^, it was not regarded as a meaningful change in BMI. Overall survival (OS) was defined as the interval from diagnosis of brain metastases to death.

### Statistical Analyses

Differences between baseline characteristics among the BMI categories were assessed using the chi-square test for categorical variables. A restricted cubic spline (RCS) was used to visualize the relationship between the BMI values and hazard ratios of all-cause mortality, followed by a cox regression model. The Kaplan–Meier curves were applied to compare the difference among the BMI categories. We estimated the adjusted-hazard ratio (adjusted-HR) and 95% confidence interval (95%CI) by the Cox regression model, and the adjusted HR considered factors including sex, age, Karnofsky performance status score, primary cancer site, the radiotherapy, target therapy, chemotherapy, and the location and number of brain metastases. The cutoff value of age was determined by the median value. These factors were reported as prognostic variables (15). We added BMI to the variables of Graded Prognostic Assessment for brain metastases (GPA: number of brain metastases, Karnofsky performance status, age, and extracranial metastases) (16) to establish a novel prediction model. The receiver operating characteristic curve (AUC) and integrated discrimination improvement (IDI) were used to assess the increased certainty provided by BMI (17). The interactions between BMI and the subgroups were assessed to identify the potential influence factors. *P*-values, were reported as two-sided and < 0.05 were considered statistical difference. All analysis was performed by R software (version 4.0.3, Vienna, Austria).

## Results

### Characteristics of Patients

A total of 2,466 patients were included in the analysis, with a median BMI of 22.39 kg/m^2^ (IQR 20.31–24.33 kg/m^2^). There were 241 in the underweight group, 1,503 in normal-weight group, and 722 in the overweight or obese group, with a median BMI of 17.53 kg/m^2^ (IQR 16.73–18.03 kg/m^2^), 21.62 kg/m^2^ (IQR 20.31–22.84 kg/m^2^), and 25.63 kg/m^2^ (IQR 24.61–27.01 kg/m^2^), respectively. The median age was 57 years (IQR 49–65 years). Among these patients, 57.5% (1,418/2,466) were men and 42.5% (1,048/2,466) were women. Compared to the underweight group, a higher proportion of male patients was found in the overweight or obese group (63% vs. 52%). In the other baseline characteristics, there was no statistical significance among the three groups (Table 1).

**TABLE 1:** Characteristics of patients.

Variable	BMI<18.5 (*n* = 241)	BMI 18.5–23.9 (*n* = 1,503)	BMI ≥ 24 (*n* = 722)	*P*-value
Sex				0.003
Female	115 (48%)	663 (44%)	270 (37%)	
Male	126 (52%)	840 (56%)	452 (63%)	
Age				0.05
<57 years	106 (44%)	769 (51%)	383 (53%)	
≥57 years	135 (56%)	734 (49%)	339 (47%)	
KPS				0.46
>70	189 (78%)	1,244 (83%)	606 (84%)	
≤70	52 (22%)	259 (17%)	116 (16%)	
Accept chemotherapy				0.11
Yes	151 (63%)	1,012 (67%)	504 (70%)	
No	90 (37%)	491 (33%)	218 (30%)	
Accept radiotherapy				0.62
Yes	103 (43%)	650 (43%)	327 (45%)	
No	138 (57%)	853 (57%)	395 (55%)	
Accept target therapy				0.85
Yes	59 (24%)	382 (25%)	176 (24%)	
No	182 (76%)	1,121 (75%)	546 (76%)	
Metastasis from lung cancer				0.65
Yes	129 (54%)	812 (54%)	375 (52%)	
No	112 (46%)	691 (46%)	347 (48%)	
The number of brain metastases				0.32
Single	55 (23%)	366 (24%)	194 (27%)	
Multiple	186 (77%)	1,137 (76%)	528 (73%)	

*BMI, body mass index (recorded when brain metastases was diagnosed); KPS, Karnofsky performance status.*

### Body Mass Index as a Prognostic Factor for Overall Survival

**FIGURE 1:**
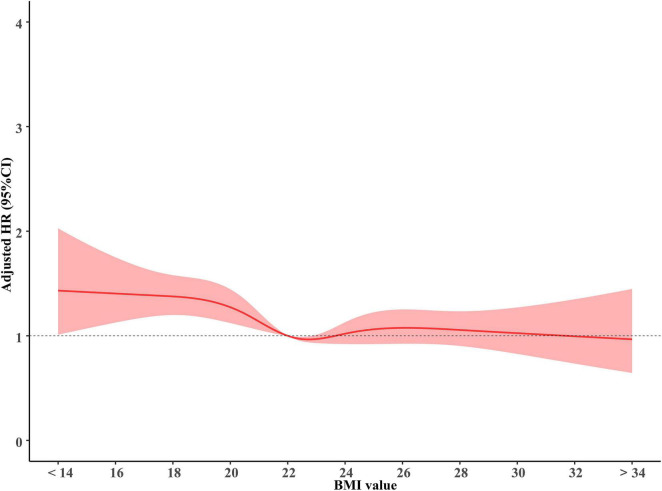
The association of BMI with overall survival. BMI, body mass index (recorded when brain metastases was diagnosed).

As shown in Figure 1, BMI was a prognostic factor for OS in brain metastases. The hazard ratios were increased for patients with a lower BMI, indicating that underweight individuals had a poorer prognosis. Notably, the BMI effect on OS was significantly non-linear on the relative hazard scale; from the BMI of approximately 22 kg/m^2^ to the highest BMI in the cohort, the hazard ratios presented insignificant differences for patients with increased BMI.

### The Impact of Body Mass Index and Weight Loss on Overall Survival

**FIGURE 2:**
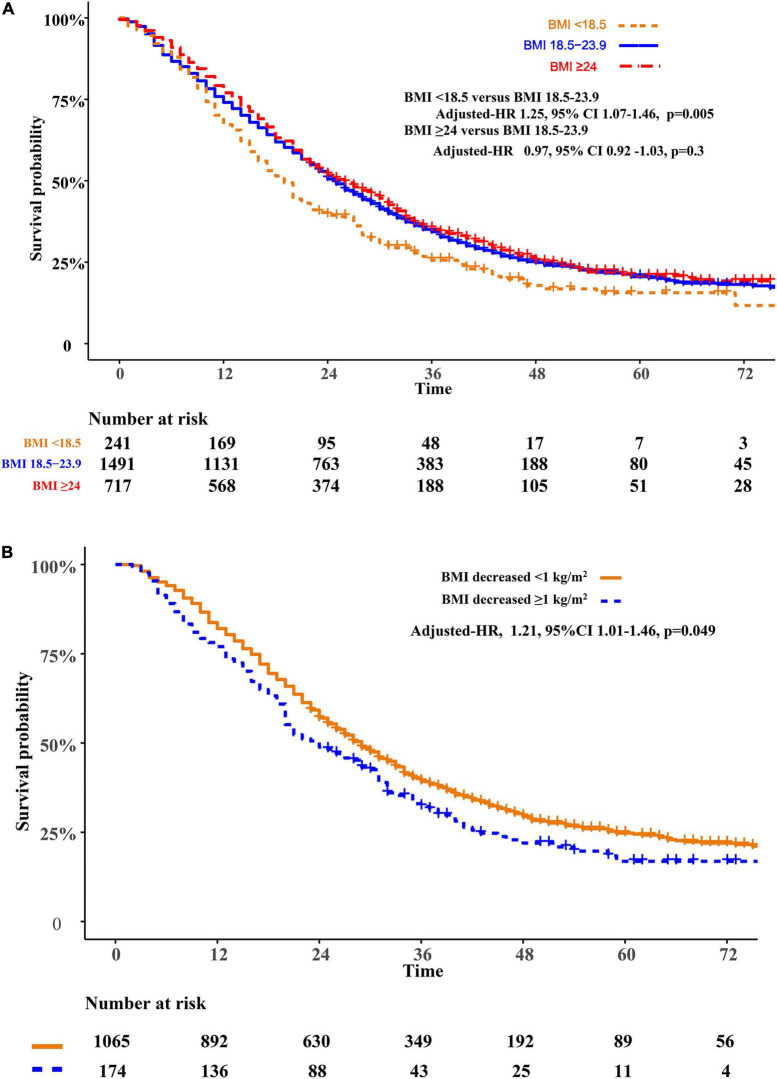
Overall survival comparisons of BMI **(A)** and weight change **(B)**. BMI, body mass index (recorded when brain metastases was diagnosed).

Relative to patients with normal weight, patients who are underweight were associated with poor prognosis (adjusted HR 1.25, 95%CI 1.07–1.46, *p* = 0.005). However, the overweight or obese group showed similar overall survival (adjusted HR 0.97, 95%CI 0.92–1.03, *p* = 0.3) when compared to the normal-weight group. Patients with weight loss were associated with a higher risk of mortality (adjusted HR 1.21, 95%CI 1.01–1.46, *p* = 0.049)compared with patients without significant weight loss (Figure 2). We modified the GPA model for brain metastasis prognosis by adding BMI information. Adding BMI to the GPA significantly improved the performance (IDI 5.2%, *p* < 0.001; AUC, *p* < 0.001) for predicting overall survival than GPA alone (Supplementary Figures 1, 2).

### Subgroup Analysis and Interaction Tests

In subgroup analysis, we found that analysis of P for interaction across each of these subgroups was insignificant not only in BMI < 18.5 kg/m^2^ vs. ≥ 18.5 kg/m^2^ (Table 2), but also in the underweight vs. the normal weight group and the overweight vs. normal weight (Supplementary Table 1). Meanwhile, subgroup analysis of patients’ metastasis from breast cancer or prostate cancer found that there were no significant differences between the overweight and the normal weight group (adjusted HR 0.91, 95%CI 0.71–1.15, *p* = 0.43; *p* for interaction was 0.39).

**TABLE 2:** Subgroup analysis.

Subgroup	Events	Total	BMI < 18.5 vs. BMI ≥ 18.5		Test for interaction
			Adjusted-HR (95%CI)	*P*-value	
Sex					0.93
Female	705	1,048	1.29 (1.02–1.62)	0.03	
Male	1,055	1,418	1.28 (1.05–1.57)	0.03	
Age					0.86
<57 years	854	1,258	1.25 (0.99–1.59)	0.06	
≥57 years	906	1,208	1.30 (1.06–1.59)	0.01	
KPS					0.91
>70	1,434	2,039	1.29 (1.09–1.53)	0.004	
≤70	326	427	1.24 (0.89–1.73)	0.2	
Accept chemotherapy					0.45
Yes	1,188	1,667	1.21 (1.01–1.47)	0.049	
No	572	799	1.33 (1.03–1.70)	0.03	
Accept radiotherapy					0.63
Yes	777	1,080	1.31 (1.04–1.65)	0.02	
No	983	1,386	1.20 (0.98–1.47)	0.08	
Accept target therapy					0.3
Yes	414	617	1.29 (0.94–1.78)	0.11	
No	1,346	1,849	1.24 (1.05–1.48)	0.01	
Accept therapy for primary cancer					0.29
Yes	1,445	2,058	1.32 (1.11–1.57)	0.001	
No	315	408	1.06 (0.76–1.49)	0.72	
Metastasis from lung cancer					0.14
Yes	960	1,316	1.16 (0.94–1.44)	0.16	
No	800	1,150	1.42 (1.14–1.78)	0.001	
The number of brain metastases					0.28
Single	386	615	1.65 (1.17–2.32)	0.004	
Multiple	1,374	1,851	1.22 (1.03–1.45)	0.02	

*BMI, body mass index (recorded when brain metastases was diagnosed); KPS, Karnofsky performance status.*

### The Correlation of Body Mass Index With Therapy Effectiveness

**FIGURE 3:**
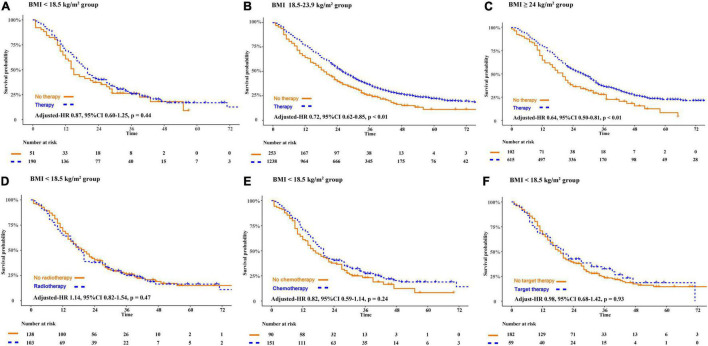
The disparity of survival outcome in patients receiving anti-cancer therapies or not according to the classification of BMI. BMI, body mass index (recorded when brain metastases was diagnosed).

Compared with patients who declined therapy, patients receiving therapy obtained a better OS in the normal weight group (adjusted-HR 0.72, 95%CI 0.62–0.85, *p* < 0.01) and the overweight or obesity group (adjusted-HR 0.64, 95%CI 0.50–0.81, *p* < 0.01). However, in patients who are underweight, there was no significant difference in OS whether they received cancer treatment or not (adjusted HR 0.87, 95%CI 0.60–1.25, *p* = 0.44). We further asked if patients who are underweight obtained a survival benefit from chemotherapy, radiotherapy, and target therapy. Compared to patients who declined therapy, there were no overall survival benefit in patients receiving radiotherapy (adjusted-HR 1.14, 95%CI 0.82–1.54, *p* = 0.47), chemotherapy (adjusted-HR 0.82, 95%CI 0.59–1.14, *p* = 0.24), or targeted therapy (adjusted-HR 0.98, 95%CI 0.68–1.42, *p* = 0.93) (Figure 3).

## Discussion

Our study showed that patients who are underweight and patients with significant weight loss both experienced an increased risk of mortality. In addition, we found that anti-cancer therapies do not significantly improve overall survival in patients who are underweight. These findings highlight the possibility of prolonging survival in patients with brain metastasis by maintaining or increasing body weight. Alternatively, since weight loss is quite common in cancer, weight may be an indicator of the disease process and may not be actionable.

A previous study indicated that BMI was strongly associated with prognosis in patients with brain metastases (11). A Swiss study based on 703 patients with brain metastases reported that high BMI was correlated with better overall survival (*p* = 0.03), and underweight with worse outcomes (*p* = 0.047) (12). It showed that the worse outcome in patients who are underweight was driven by those with primary lung cancer (*p* = 0.005), and that there was no difference between the patients who are underweight and the patients with normal weight in other types of cancer (*p* = 0.87) (12). However, that study included only 50 cases in the underweight group, and a biased estimate may have occurred due to the small dataset.

Some of the conflicting results from previous reports may be due to the different cancer types studied. For example, underweight patients (BMI < 18.50 kg/m^2^) had higher mortality (HR 1.61, 95% CI 1.53–1.70, *p* < 0.0001) compared with patients with normal weight (BMI 18.50–24.99 kg/m^2^) in colorectal cancer (18, 19). However, Troeschel et al. suggested that obesity at diagnosis (HR 1.23, 95% CI 1.11–1.35) and weight gain (HR 1.27, 95% CI 1.12–1.45) after a prostate cancer diagnosis may be associated with higher all-cause mortality (20). In breast cancer, overweight or obesity has a negative impact on the effectiveness of neoadjuvant chemotherapy (21). In our study population, we found no differences in overall survival between patients who are overweight and normal weight, with brain metastasis from different cancers, including lung cancer (test for interaction: *p* = 0.31), breast cancer, or prostate cancer (test for interaction: *p* = 0.39). It should be noted that there are differences in baseline BMI between our study population and those from other studies; the proportion of obese individuals in this cohort is lower.

Among patients who are underweight, we found no significant differences in OS for patients receiving chemotherapy, radiotherapy, or targeted therapy compared to those who declined therapy. A tangentially relevant study was a meta-analysis involving 3,768 individual patients with cancer treated with immune checkpoint inhibitors, where the median OS was significantly higher in overweight or in patients with obesity than in patients who are not overweight (20.7 vs. 11.3 months, *p* < 0.001) (22). It remains unclear whether an optimal combination of cancer therapy and nutrition may provide benefits for underweight patients.

Some limitations in this study should be acknowledged. The retrospective nature of our study from a single institution may potentially affect results. Firstly, we could not collect the information about the disease course of primary cancer and the treatment for primary cancer before brain metastases were diagnosed. It is unknown whether low BMI itself puts patients at risk of disease progression or is an indicator of disease progression, or whether the treatment process of primary cancer resulted in low BMI and heightened the risk for mortality. Second, the limited number of patients (*n* = 241) in the underweight group place limits on the statistical power. Additionally, in the subgroup analysis of breast cancer or prostate cancer, we could not compare the underweight with the normal-weight group, because only six patients with brain metastases from breast cancer or prostate cancer were underweight. Finally, we were unable to adjust for some underlying diseases in our analysis due to missing data, which could affect our results. For example, overweight individuals have a disposition for diabetes, but diabetes was associated with an increased risk of cancer-related mortality (23, 24).

## Conclusion

Underweight and significant weight loss is associated with poor prognosis in brain metastases. Meanwhile, anti-cancer therapies do not significantly improve overall survival of patients who are underweight. This suggests the importance of maintaining body weight and nutrition in patients with brain metastases.

## Data Availability Statement

The raw data supporting the conclusions of this article will be made available by the authors, without undue reservation.

## Ethics Statement

The studies involving human participants were reviewed and approved by the West China Hospital of Sichuan University. Written informed consent for participation was not required for this study in accordance with the national legislation and the institutional requirements.

## Author Contributions

YH, YZ, and FF: study concept and design. YH, YZ, YP, RZ, ZL, and JY: acquisition and interpretation of the data. YH and YZ: drafting of the manuscript and statistical analysis. WC, FF, and XP: critical revision of the manuscript. FF and XP: administrative and technical support. All authors final approval of the manuscript.

## Conflict of Interest

The authors declare that the research was conducted in the absence of any commercial or financial relationships that could be construed as a potential conflict of interest.

## Publisher’s Note

All claims expressed in this article are solely those of the authors and do not necessarily represent those of their affiliated organizations, or those of the publisher, the editors and the reviewers. Any product that may be evaluated in this article, or claim that may be made by its manufacturer, is not guaranteed or endorsed by the publisher.

## Supplementary Material

Supplementary Figure 1 Area under receiver operating characteristic curve (AUC) to assess the increased certainty provided by BMI. GPA, Graded Prognostic Assessment for brain metastases; BMI, body mass index (recorded when brain metastases was diagnosed).

Supplementary Figure 2 The modified-GPA model for brain metastasis prognosis by adding BMI information. BMI, body mass index (recorded when brain metastases was diagnosed); KPS, Karnofsky performance status.
