# Supplementary material for: Association of Underweight and Weight Loss With Poor Prognosis and Poor Therapy Effectiveness in Brain Metastases: A Retrospective Study
Source: Front Nutr. 2022 Jul 1;9:851629. doi: 10.3389/fnut.2022.851629 (PMC9286517; doi:10.3389/fnut.2022.851629)
Supplement: Supplementary file 1 [file Table_1.DOCX]

Table S1. Subgroup analysis of underweight versus normal weight group and the overweight versus normal weight.

| **Subgroup** | **BMI＜18.5 versus BMI 18.5-23.9** | | **Test for**  **interaction** | **BMI≥24 versus BMI 18.5-23.9** | | **Test for**  **interaction** |
| --- | --- | --- | --- | --- | --- | --- |
|  | **Adjusted-HR (95%CI)** | **P value** |  | **Adjusted-HR (95%CI)** | **P value** |  |
| **Sex** |  |  | 0.84 |  |  | 0.60 |
| Female | 1.28 (1.01-1.61) | 0.04 |  | 0.99 (0.91-1.08) | 0.84 |  |
| Male | 1.25 (1.02-1.55) | 0.03 |  | 0.97 (0.91-1.04) | 0.37 |  |
| **Age** |  |  | 0.95 |  |  | 0.70 |
| < 57 years | 1.22 (0.96-1.56) | 0.1 |  | 0.98 (0.91-1.06) | 0.57 |  |
| ≥ 57 years | 1.25 (1.02-1.54) | 0.03 |  | 0.96 (0.89-1.03) | 0.28 |  |
| **KPS** |  |  | 0.99 |  |  | 0.83 |
| >70 | 1.25 (1.05-1.49) | 0.01 |  | 0.97 (0.91-1.02) | 0.24 |  |
| ≤70 | 1.25 (0.88-1.76) | 0.2 |  | 1.02 (0.89-1.15) | 0.8 |  |
| **Accept chemotherapy** | |  | 0.68 |  |  | 0.16 |
| Yes | 1.21 (0.99-1.47) | 0.06 |  | 0.99 (0.94-1.06) | 0.96 |  |
| No | 1.27 (0.98-1.65) | 0.07 |  | 0.93 (0.84-1.03) | 0.15 |  |
| **Accept radiotherapy** | |  | 0.80 |  |  | 0.30 |
| Yes | 1.26 (0.99-1.59) | 0.06 |  | 0.94 (0.87-1.02) | 0.16 |  |
| No | 1.20 (0.97-1.48) | 0.09 |  | 1.00 (0.93-1.08) | 0.93 |  |
| **Accept target therapy** | |  | 0.86 |  |  | 0.15 |
| Yes | 1.20 (0.87-1.67) | 0.26 |  | 0.92 (0.82-1.03) | 0.13 |  |
| No | 1.23 (1.03-1.47) | 0.02 |  | 0.99 (0.93-1.05) | 0.81 |  |
| **Primary cancer site** | |  | 0.30 |  |  | 0.09 |
| Lung cancer | 1.16 (0.94-1.44) | 0.17 |  | 1.01 (0.94-1.09) | 0.72 |  |
| Others | 1.34 (1.07-1.69) | 0.01 |  | 0.93 (0.86-1.01) | 0.07 |  |
| **The number of brain metastases** | | | 0.18 |  |  | 0.13 |
| Single | 1.69 (1.18-2.41) | 0.004 |  | 1.05 (0.94-1.17) | 0.37 |  |
| Multiple | 1.18 (0.99-1.41) | 0.06 |  | 0.95 (0.89-1.01) | 0.14 |  |

BMI, body mass index (recorded when brain metastases was diagnosed); KPS, Karnofsky performance status.
