# Supplementary figures and images for: Association of Underweight and Weight Loss With Poor Prognosis and Poor Therapy Effectiveness in Brain Metastases: A Retrospective Study
Source: Front Nutr. 2022 Jul 1;9:851629. doi: 10.3389/fnut.2022.851629 (PMC9286517; doi:10.3389/fnut.2022.851629)

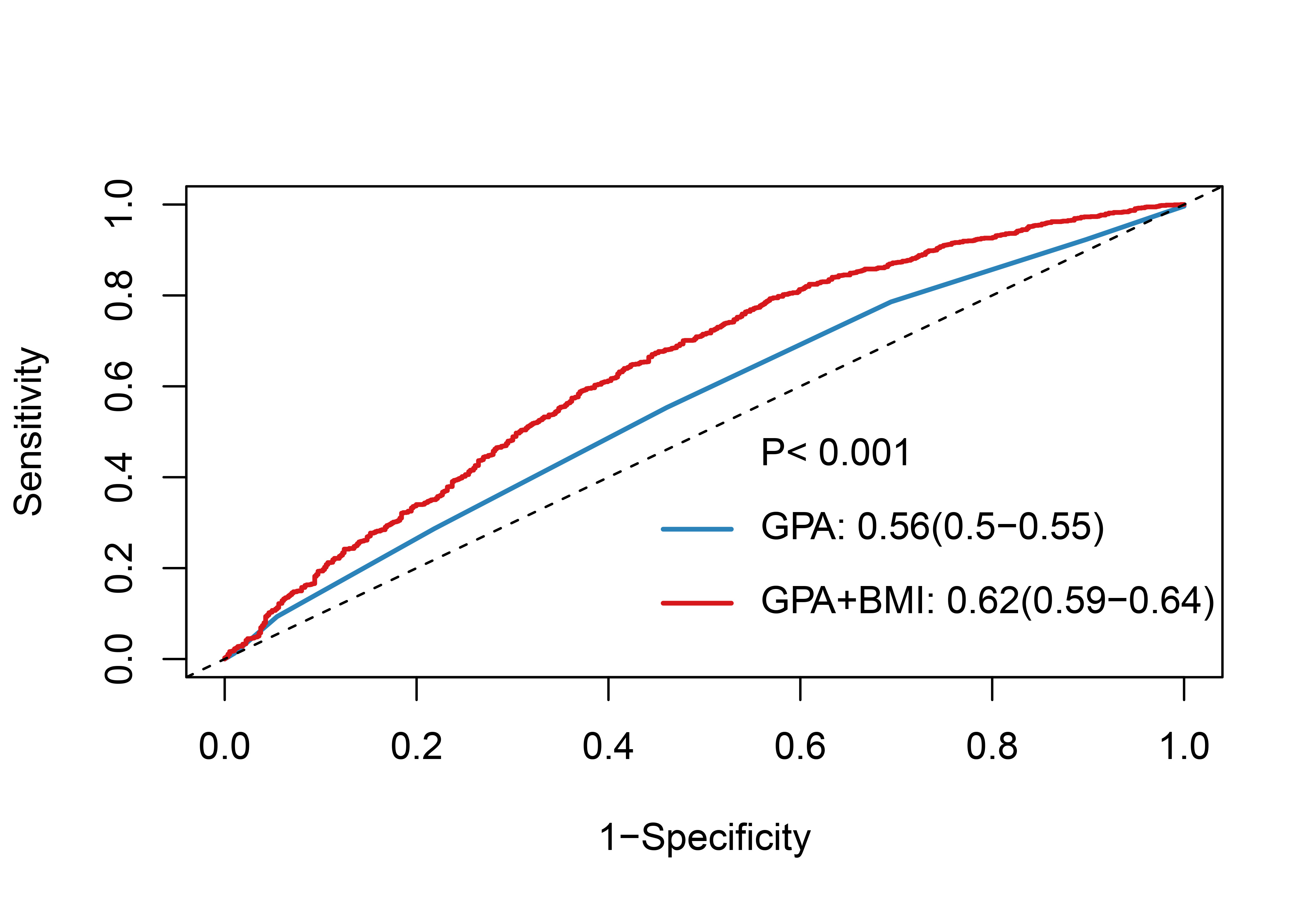

Supplement: Supplementary Figure 1 — Area under receiver operating characteristic curve (AUC) to assess the increased certainty provided by BMI. GPA, Graded Prognostic Assessment for brain metastases; BMI, body mass index (recorded when brain metastases was diagnosed). [file Image_1.JPEG]

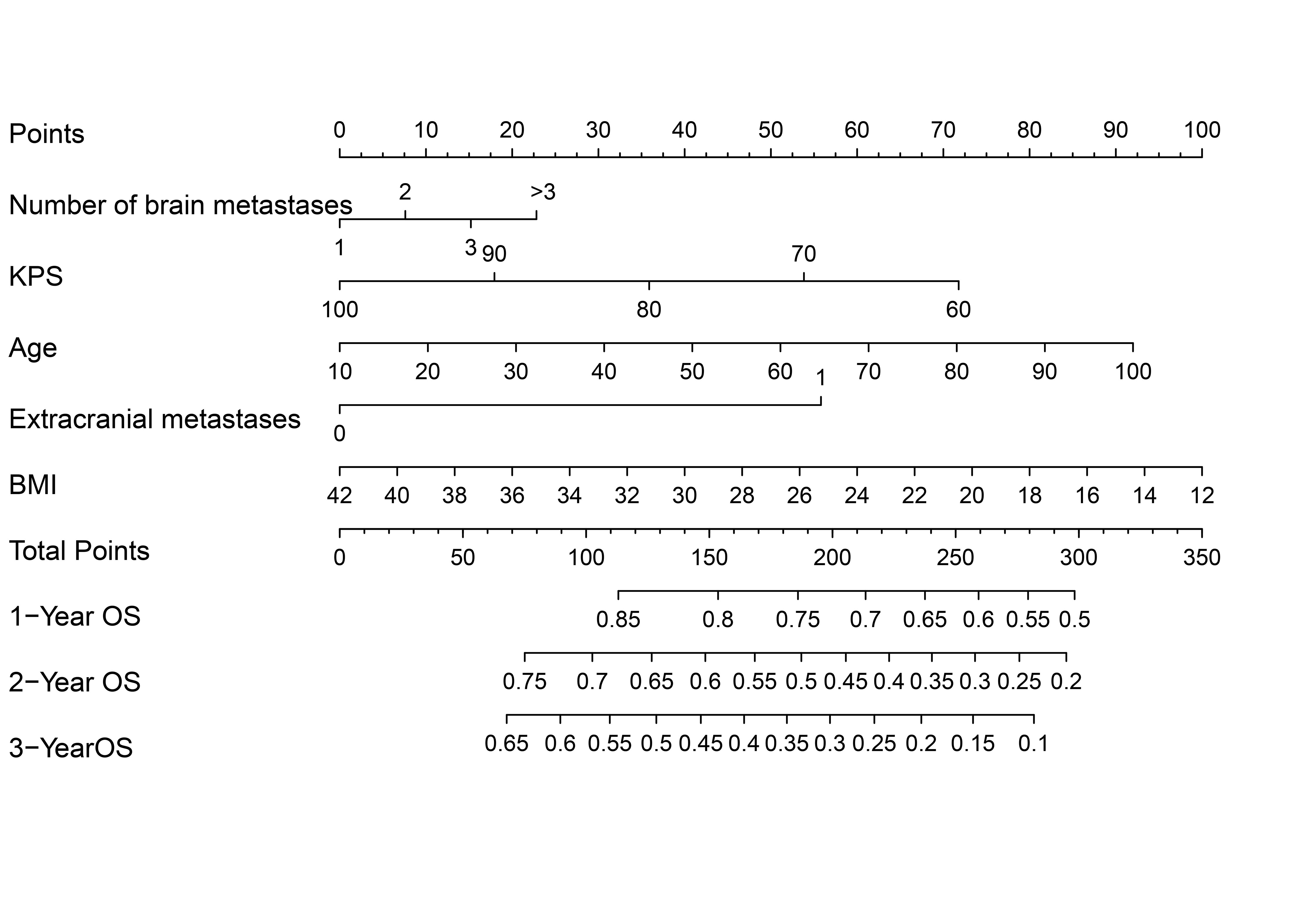

Supplement: Supplementary Figure 2 — The modified-GPA model for brain metastasis prognosis by adding BMI information. BMI, body mass index (recorded when brain metastases was diagnosed); KPS, Karnofsky performance status. [file Image_2.JPEG]
